# The British Pharmaceutical Codex: An Imperial Dispensatory

**Published:** 1907-12

**Authors:** 


**The British Pharmaceutical Codex: An Imperial Dispensatory.** Pp. x., 1,422. London: The Pharmaceutical Society. 1907.

The second title of this work has been placed first as it most aptly sums up its function.

The word Imperial may well be used, for the *Codex* includes monographs not only on the drugs now official in the B.P., but also those used in our colonies and dependencies the world over. Thus while, of course, Calumba and Chirata will be found in alphabetical order, so also appear Coscinium and Andrographis for the benefit of the practitioner in India and the Malay Archipelago. The book is issued by the authority of the Pharmaceutical Society of Great Britain, and is the work, during the last three years, of a committee including the leading pharmacists of Great Britain, while Dr. W. E. Dixon, the Professor of Pharmacology at King's College, Oxford, has arranged the concise notes on the Physiological Action and on Prescribing.

The book is intended to meet three great wants not met by the British Pharmacopœia.

First of all it includes a large number of drugs and preparations which, while in every-day use, have not yet been considered worthy of a place in the B.P. A national pharmacopœia is almost necessarily conservative ; this dispensatory is liberal to a degree in its admission of new—relatively new—drugs and preparations. Examples of new drugs included are cacodylic, formic and glycerophosphoric acids, salts of these acids, and preparations made from them. In this category adrenine and three liquors prepared from it will certainly attract notice, in view of the widespread use of preparations of the adrenal glands, and the difficulty of knowing what is the best preparation to use. Emulsio chloroformi will appeal to the medico who does his own dispensing, by economising his use of alcohol for making spirit of chloroform. Emulsio magnesiæ, which will be better known by its synonym of lac magnesiæ, is wanted every day as an antacid, and also as a mouth wash. The old concentrated infusions—strength 1–7—we find here, and will quite obviate the use of the rather clumsy concentrated liquors of the B.P. Solutio saponis ætherea will enable the member of a hospital staff to obtain the same ether soap for his bag that he uses at the hospital.

The second innovation made by the *Codex* is that of introducing absolutely new classes of preparations, characteristic of elegant pharmacy, and also of general medical and surgical interest. Balnea, gelatin capsules, carbasus (gauze) plain and medicated, will enable the surgeon to order, and the pharmacist to supply, articles of known and standard quality ; lints, elixirs which will obviate the resort to American dispensatories for elegance of compounding ; gargles, gelatines, guttæ. insufflationes, linctus, misturæ, nebulæ, parogens, tabellæ, tablettæ, will, it is safe to prophesy, go far towards rendering uniform the hospital pharmacopœias of the future, in which it will be possible to include mist. gentian acid, B.P.C., the due inclusion of the last three letters indicating the source of the formula.

The third function which this work is destined to achieve may in many respects, and possibly from the standpoint of the prescriber in all respects, be the most important. Up to the present every post encumbers the prescriber's table, and later his waste-paper basket, with more or less plausibly-worded advertisements of "new drugs," mostly chemicals of the organic type. In this flood of patented nostrums it is almost impossible to separate good from bad, and even when conviction of the value of a drug or its preparation is reached, one is confronted with the necessity of prescribing a patent article more or less "secret" in its nature. Add to this that many drugs of this nature—practically identical in composition—appear under a number of different patent names, and the unsatisfactory position of the prescriber is emphasised. For example, hexamethylenetetramine may be prescribed as aminoform, ammonio-formaldehyde, ammonaldehyde, cystamine, cystogen, formin, metramine, urisol, uritone, urotropine, and vesalvine (one of these names at least being of Bristol origin). In cases such as this the compilers have coined a name for the drug—formamine in this case—indicated its formula and characteristics, and inserted at the end of the monograph a list of patent names under which it is known. Further examples are acetannin, tannigen, methyl ditannin, tannoform, sodii anilarsenas, atoxyl, &c., &c. The gain to prescribers in ordering such drugs under their *Codex* names will be threefold: first, they will be prescribing a drug of whose nature and composition they are aware, they will discourage the introduction and use of quack nostrums and secret remedies, and they will in many cases place remedies within the reach of patients who could not afford to pay the fancy prices at which many fancy patent drugs are sold.

